# Impact of lifestyle factors on dietary vitamin B_6_ intake and plasma pyridoxal 5′-phosphate level in UK adults: National Diet and Nutrition Survey Rolling Programme (NDNS) (2008–2017)

**DOI:** 10.1017/S0007114523000417

**Published:** 2023-10-28

**Authors:** Asrar Alsaeedi, Simon Welham, Peter Rose

**Affiliations:** Division of Food, Nutrition and Dietetics, School of Biosciences, University of Nottingham, Loughborough, Leicestershire LE12 5RD, UK

**Keywords:** Vitamin B_6_, PLP, Lifestyle, Medication, Elderly, Nutrition

## Abstract

Reduction in dietary vitamin B_6_ intake is associated with an increased relative risk of diseases such as cancer, atherosclerosis and cognitive dysfunction. The current research has assessed vitamin B_6_ intakes and PLP concentrations as a marker of vitamin B_6_ status among the UK adult (≥ 19 years) population. This study was carried out using a cross-sectional analysis of the National Diet and Nutrition Survey Rolling Programme (NDNS) (2008–2017). The impacts of lifestyle factors, including type of diet, smoking, alcohol consumption, and commonly used medications grouped by therapeutic usage, were determined, and data were analysed using IBM SPSS^®^. Results are expressed as medians (25th–75th percentiles), with *P* values ≤ 0·05 considered statistically significant. Among UK adults, the median intakes of total population of dietary vitamin B_6_ met the reference nutrient intake and median plasma PLP concentrations were above the cut-off of vitamin B_6_ deficiency; however, we found an association between reduction in vitamin B_6_ intake and plasma PLP concentration and age group (*P* < 0·001). Smokers had significantly lower plasma PLP concentrations than non-smokers (*P* < 0·001). Moreover, regression analysis showed some commonly used medications were associated with plasma PLP levels reduction (*P* < 0·05). Taken together, we report on a tendency for dietary vitamin B_6_ intake and plasma PLP concentrations to decrease with age and lifestyle factors such as smoking and medication usage. This information could have important implications for smokers and in the elderly population using multiple medications (polypharmacy).

The B_6_ vitamins comprise three phosphorylated pyridine derivatives represented by pyridoxal 5′-phosphate (PLP), pyridoxamine 5′-phosphate and pyridoxine 5′-phosphate^(1)^. These derivatives are obtained from a diverse array of food items, including meat, milk products, beans, nuts, potatoes and several fruits and vegetables^(2)^. Following ingestion, these molecules undergo dephosphorylation by intestinal phosphatases, absorbed and then converted in the liver to the active form of vitamin B_6_, PLP. PLP serves as a cofactor for more than 140 enzymes and is therefore a critical component of many biochemical pathways^(3)^. It has roles in the immune system^(4)^, neurotransmission and brain function^(2)^, synthesis of metabolic products like lipids and amino acids^(5)^, as a modulator of transcription factors^(6)^ and in redox systems^(7)^. PLP is considered as one of the most sensitive markers for vitamin B_6_ status in humans^(8)^. In recent times, lifestyle factors such as smoking^(9)^, alcohol consumption^(10)^ drug consumption^(11)^ and differing dietary patterns such as vegetarianism^(12)^ have been reported to be key drivers of low dietary vitamin B_6_ intake or PLP concentration reduction. Indeed, it is now widely recognised that diminished dietary vitamin B_6_ intakes correlate with CVD^(13)^, Parkinson’s disease^(14)^ and some cancers^(15)^. Moreover, increased evidence points to stronger associations of PLP level reduction with increased risk of CVD including stroke^(16)^, irritable bowel syndrome^(17)^, thrombosis^(18)^, severity of COVID-19 infection^(19)^ and diabetes^(20)^. Importantly, PLP level reduction can be caused by certain medical procedures and genetic factors, for example, in renal transplant recipients^(21)^, adult intestinal transplant recipients^(22)^ and in those suffering seizures^(23)^. These links correspond with the known functional roles of PLP in many enzymes catalysed reactions important in the immune system^(4)^, trans-methylation^(24)^ and trans-sulphuration pathways^(25)^. Nutrient deficiencies are of a global health concern^(26)^ due to their associations with many chronic health conditions^(27)^. Reduction in dietary vitamin B_6_ intake is bolstered by ageing^(28)^, rates of undernutrition^(29)^ and smoking^(30)^. Moreover, the usage of medications may also be an important driver of deficiency^(11)^. Worldwide the number of drug prescriptions has increased, particularly in developed countries^(31)^. Indeed, in the UK alone, the number of drug prescriptions increased by 65% between 1999 and 2009^(32)^. Moreover, in England, among elderly populations (65^+^), the number of individuals taking five or more types of medications has increased from 12 % to 49 % in the last two decades^(33)^. In view of the widespread use of prescription drugs, the current work seeks to assess lifestyle factors and medication usage on dietary vitamin B_6_ intake and plasma PLP concentration in the general UK adult population (≥ 19 years) using the National Diet and Nutrition Survey Rolling Programme (NDNS-RP; 2008–2017). Only a limited number of studies have explored how lifestyle factors impact on vitamin B_6_ intake and PLP levels in humans. Therefore, the current research utilised the NDNS dataset to establish dietary vitamin B_6_ and plasma PLP concentrations and assessed how a wide range of lifestyle factors and medications influence the levels of these important nutrients in humans.

## Materials and methods

### The National Diet and Nutrition Survey Rolling Programme

NDNS-RP is a national cross-sectional survey to assess diet, nutrient intake and nutritional status of the UK population aged 1·5 years and above, who live in a private household. Data from the NDNS were obtained from the UK Data Service (https://www.ukdataservice.ac.uk/). Detailed methodology is reported in the user guide for UK data and elsewhere^(34)^; however, briefly, to produce representative data of the UK population, this survey was carried out in all four UK countries: England, Scotland, Wales and Northern Ireland. The survey was designed to recruit 1000 subjects annually (500 children (1·5 years to 18 years) and 500 adults (19 years and above)), with a boost sample of an additional 600 subjects. Sample selection was conducted randomly from the Postcode Address File, a list of all UK addresses. These addresses were then clustered into primary sampling units, smaller geographical areas based on postcode sectors, to enhance cost-effectiveness. From each primary sampling unit, a list of addresses was selected randomly. To reach this sample, trained interviewers contacted the addresses to enrol the participants, based on two stages, namely interviewer (first) and nurse (second) stages. In the current study, the most recent timepoint in NDNS when we started the work was 2017. As such, the individual and personal level dietary data from all years (2008–2017) were joined by one researcher S.W. in SPSS file form. Both male and female aged 19 years and above were included in the current work.

### First stage: dietary data, smoking and alcohol consumption

During the initial visit by trained fieldworkers, a face-to-face computer-assisted personal interview was performed with each participant. Dietary data were obtained by using four days’ estimated food diaries that were explained to the participants. In year 1 of the study, dietary recording was always started on Thursday, Friday or Saturday and included both weekend days, while in year 2, the study was designed to over-represent weekdays than weekends. From year 3 onwards, dietary recording was started on any day of the week for four consecutive days (to evenly represent all weekdays). Briefly, participants were asked to report foods and drinks that were consumed, both in and outside the home, during the 4 days: including brand name, portion size and recipe if home-cooked. Household measures (e.g. 4 teaspoons of peanut butter) or weights reported on labels (e.g. 200 ml can of lemonade) were used to estimate portion sizes. Then, each food or drink item was assigned a code and dietary assessment was performed using a platform called Diet In Nutrients Out (DINO) – developed at the Medical Research Council (MRC) Elsie Widdowson laboratory (MRC EWL) – that is based on Public Health England’s NDNS Nutrient Databank food composition data. In fact, this database is updated annually by the Food Standards Agency to revise the existing food codes or remove foods that are no longer sold in the UK. According to the British Nutrition Foundation, the reference nutrient intake (RNI) for dietary vitamin B_6_ intake for adults aged 19 years and above is 1·4 mg/d for male and 1·2 mg/d for female. In the current study, participants who used supplementation were excluded.

During the interviewer stage, types of diet was collected from participants by an interview and defined as omnivore or vegetarian or vegan. Additionally, smoking status was also assessed and collected by self-completion questionnaires; in this study, the number of cigarettes per day was defined as follows: non-smokers or 1–5 cigarettes/d or 6–9 cigarettes/d or ≥ 10 cigarettes/d. Moreover, drinking habit – collected by self-completion questionnaires – was categorised as follows: ((non-alcoholic or ≤ 4 units/d (men) and ≤ 3 units/d (women)) or (> 4 units/d and ≤ 8 units/d (men), > 3 units/d and ≤ 6 units/d (women)) or (> 8 units/d units (men) and > 6 units/d units (women))).

Height and weight were measured using a portable stadiometer, and BMI (kg/m^2^) was calculated as weight (kg) divided by height (m) squared. Only participants with three or four completed days of the food diary were invited to the second stage of the survey to participate in blood measurements.

### Second stage: laboratory measurements and medication use

Participants who took part in computer-assisted personal interview and completed at least three dietary records were asked to be involved in the second stage of the surveys, which took the form of a nurse visit – to take blood samples in participants’ homes by trained nurses. The nurse stage was carried out within 2–4 months of the final reviewer visit. Following overnight fasting, blood samples were obtained by venepuncture-trained nurses or phlebotomists and stored in a cool box (at approximately 4°C). Samples were delivered to a locally recruited laboratory for prompt analysis within 2 hours of collection. Then, samples were processed and stored at −40°C before transporting on dry ice and delivering to MRC EWL, where samples were stored frozen at −80°C until analysis was conducted.

Since all dietary vitamin B_6_ forms are converted to PLP, PLP levels were assessed in this study. PLP concentration in plasma was measured with a reverse-phase HPLC method with post-column derivatisation and fluorometric detection as described^(35)^. In this work, the cut-off of vitamin B_6_ deficiency is < 20 nmol/l of plasma PLP concentration, while 20–<30 nmol/l is indicative of marginal deficiency^(36,37)^. Furthermore, during this visit, prescribed medication use information was collected. However, no details were reported regarding the duration and doses of the used medications

### Ethics

The NDNS was conducted according to the guidelines laid down in the Declaration of Helsinki. The ethical approval of all procedures including human subjects was obtained from the Oxfordshire A Research Ethics Committee for the NDNSD-RP 2008–2013 (Ref. No. 07/H0604/113), while for the NDNS-PR 2014–2017, the approval was obtained from the Cambridge South NRES Committee (Ref. No. 13/EE/0016). The study is registered with the ISRTCN registry as ISRCTN17261407 (https://www.isrctn.com/ISRCTN17261407). Written informed consent was obtained from all participants.

### Data analysis

Analyses were performed in IBM SPSS^®^ Statistics for Windows, Version 27. Since the data were not normally distributed, data presented as median values and 25th–75th percentiles (*Max* and *Min* values were also reported) or numbers and percentages. Total sample dietary vitamin B_6_ intakes and plasma PLP concentrations were analysed by sex and age group – stratified by decades. Mann–Whitney *U*-test was used to compare differences between two categorical data. For more than two covariates comparison, Kruskal–Wallis H with post-hoc Tukey test was used. In GenStat 22nd edition, linear regression analysis was used to investigate associations between dietary vitamin B_6_ intakes and plasma PLP concentrations, taking into consideration age group and sex. Plasma PLP concentration was log_10_ transformed for normalisation and subject to multiple linear regression analyses to test associations between plasma PLP concentration/dietary vitamin B_6_, drugs and lifestyle factors. *P* values ≤ 0·05 were considered statistically significant throughout the study.

## Results

### Study population characteristics

From the NDNS cohort, 6802 participants aged ≥ 19 years were included in this study. The demographic characteristics of participants are presented in Table 1. Median (25th–75th percentile) age of the total population was 49 (36–63), with a higher percentage of female respondents than male noted in the current work (59 % and 41%, respectively). Median (25th–75th percentile) BMI of the total population was 27 (24–31) kg/m^2^, with 29% of total population categorised as obese (BMI ≥ 30 kg/m^2^) and 38% overweight (BMI = 25–29·9 kg/m^2^) (Table 1). However, not all participants reported BMI. Participant’s characteristics regarding lifestyle factors, including smoking, alcohol consumption, type of diet and medication usage, are summarised in Table 1. For data focussed on medication, twelve therapeutic drugs were reported in the NDNS data set (Table 1).

**Table 1.** Characteristics of UK adults aged 19 and above from NDNS, 2008–2017

			Type of diet						Smoking (cigarette/d)						Alcohol (unit/d)					
	Total		Omnivore		Vegetarian		Vegan		1–5		6−9		≥ 0		≤4 (men), ≤ 3 (women)		>4 and ≤ 8 (men), > 3 and ≤ 6 (women)		> 8 (men), > 6 units (women)	
	*n*	%	*n*	%	*n*	%	*n*	%	*n*	%	*n*	%	*n*	%	*n*	%	*n*	%	*n*	%
Number of participants (%)	6802	100 %	6636	97·6 %	157	2·3 %	9	0·1 %	249	17·7 %	137	9·7 %	1021	72·6 %	1727	42·4 %	1108	27·2 %	1239	30·4 %
	Median	25th–75th percentiles	Median	25th–75th percentiles	Median	25th–75th percentiles	Median	25th–75th percentiles	Median	25th–75th percentiles	Median	25th–75th percentiles	Median	25th–75th percentiles	Median	25th–75th percentiles	Median	25th–75th percentiles	Median	25th–75th percentiles
Median (25th–75th percentiles) of age	49 *Min*:19 *Max*:96	36–63	49 *Min*: 19 *Max*: 96	36–64	45 *Min*: 19 *Max*: 84	36–57	52 *Min*: 28 *Max*: 78	35–64	38 *Min*:19 *Max*:84	29–53	40 *Min*:19 *Max*:86	28–55	44 *Min*:19 *Max*:90	33–54	55 *Min*: 19 *Max*:93	40–69	48 *Min*:19 *Max*:87	37–59	43 *Min*:19 *Max*:87	32–53
Median (25th–75th percentiles) of BMI (kg/m^2^)	27 *Min*:15 *Max*:53	24–31	27 *Min*:15 *Max*:53	24–31	26 *Min*:18 *Max*:40	24–28	26 *Min*:17 *Max*:36	19–33	25 *Min*:17 *Max*:53	23–29	25 *Min*:18 *Max*:49	21–31	27 *Min*:15 *Max*:41	23–30	27 *Min*:15 *Max*:44	24–30	27 *Min*:18 *Max*:45	24–30	27 *Min*:17 *Max*:47	23–31
BMI classification	*n*	%	*n*	%	*n*	%	*n*	%	*n*	%	*n*	%	*n*	%	*n*	%	*n*	%	*n*	%
Underweight	24	1 %	22	1 %	1	2 %	1	17 %	3	5 %	1	3 %	3	1 %	8	2 %	2	1 %	1	1 %
Normal	590	32 %	568	32 %	21	43 %	1	17 %	26	39 %	18	47 %	76	33 %	145	31 %	92	32 %	106	32 %
Overweight	706	38 %	686	38 %	18	37 %	2	33 %	25	38 %	8	21 %	89	39 %	192	42 %	118	41 %	125	38 %
Obese	539	29 %	528	29 %	9	18 %	2	33 %	12	18 %	11	29 %	62	27 %	116	25 %	74	26 %	95	29 %
Sex																				
Number of males (%)	2810	41 %	2764	42 %	45	29 %	1	11 %	95	38 %	59	43 %	456	45 %	750	43 %	466	42 %	667	54 %
Number of females (%)	3992	59 %	3872	58 %	112	71 %	8	89 %	154	62 %	78	57 %	565	55 %	977	57 %	642	58 %	572	46 %
Region																				
England	3905	57 %	3793	57 %	106	67 %	6	67 %	161	65 %	83	60 %	497	49 %	1075	62 %	661	60 %	670	54 %
Scotland	1073	16 %	1053	16 %	20	13 %	0		27	11 %	22	16 %	202	20 %	236	14 %	170	15 %	220	18 %
Wales	952	14 %	926	14 %	23	15 %	3	33 %	28	11 %	16	12 %	144	14 %	266	15 %	142	13 %	176	14 %
Northern Ireland	872	13 %	864	13 %	8	5 %	0		33	13 %	16	12 %	178	17 %	150	9 %	135	12 %	173	14 %

	Drug use																							
	Analgesic		Antibacterial		Antidiabetic		Antidepressant		ACE inhibitor		Beta blocker		Ca blocker		Diuretic		Antiplatelet		Asthma prescribed		Lipid lowering		PPI	
	*n*	%	*n*	%	*n*	%	*n*	%	*n*	%	*n*	%	*n*	%	*n*	%	*n*	%	*n*	%	*n*	%	*n*	%
Number of participant (%)	194	13 %	26	2 %	89	6 %	185	13 %	309	12 %	173	7 %	199	8 %	188	8 %	107	7 %	114	8 %	405	16 %	184	13 %
	Median	25th–75th percentiles	Median	25th–75th percentiles	Median	25th–75th percentiles	Median	25th–75th percentiles	Median	25th–75th percentiles	Median	25th–75th percentiles	Median	25th–75th percentiles	Median	25th–75th percentiles	Median	25th–75th percentiles	Median	25th–75th percentiles	Median	25th–75th percentiles	Median	25th–75th percentiles
Median (25th–75th percentiles) of age	63 *Min*:20 *Max*:95	51–72	59 *Min*:19 *Max*:89	45–70	64 *Min*:19 *Max*:93	47–72	54 *Min*:19 *Max*:94	44–66	67 *Min*:26 *Max*:93	57–74	70 *Min*:24 *Max*:92	59–78	67 *Min*:38 *Max*:93	59–75	72 *Min*:39 *Max*:94	63–79	72 *Min*:41 *Max*:95	64–79	57 *Min*:19 *Max*:95	44–69	68 *Min*:36 *Max*:95	60–75	67 *Min*:24 *Max*:95	55–76
Median (25th–75th percentiles) of BMI (kg/m^2^)	29 *Min*:15 *Max*:48	25–33	28 *Min*:20 *Max*:38	25–32	31 *Min*:18 *Max*:46	27–36	28 *Min*:18 *Max*:48	25–33	29 *Min*:19 *Max*:47	26–33	29 *Min*:17 *Max*:46	27–33	30 *Min*:18 *Max*:48	26–33	30 *Min*:15 *Max*:48	27–34	29 *Min*:17 *Max*:47	25–32	28 *Min*:15 *Max*:49	25–31	29 *Min*:17 *Max*:46	26–33	28 *Min*:15 *Max*:47	26–31
BMI classification																								
Underweight	1	1 %	0	0 %	1	1 %	1	1 %		0 %	1	1 %	1	1 %	1	1·1 %	2	2 %	1	1 %	1	1 %	2	1 %
Normal	36	21 %	3	14 %	10	12 %	33	20 %	32	17 %	12	13 %	20	18 %	11	12·2 %	18	18 %	24	24 %	35	17 %	30	19 %
Overweight	63	37 %	10	45 %	27	34 %	67	41 %	70	37 %	36	38 %	40	36 %	31	34·4 %	33	34 %	43	42 %	76	36 %	72	45 %
Obese	69	41 %	9	41 %	42	53 %	63	38 %	87	46 %	45	48 %	49	45 %	47	52·2 %	45	46 %	34	33 %	97	46 %	56	35 %
Sex																								
Number of males (%)	65	34 %	8	31 %	44	49 %	48	26 %	155	50 %	87	50 %	93	47 %	76	40 %	56	52 %	47	41 %	215	53 %	71	39 %
Number of females (%)	129	66 %	18	69 %	45	51 %	137	74 %	154	50 %	86	50 %	106	53 %	112	60 %	51	48 %	67	59 %	190	47 %	113	61 %

	Drug use																							
	Analgesic		Antibacterial		Antidiabetic		Antidepressant		ACE inhibitor		Beta blocker		Ca blocker		Diuretic		Antiplatelet		Asthma prescribed		Lipid lowering		PPI	
	*n*	%	*n*	%	*n*	%	*n*	%	*n*	%	*n*	%	*n*	%	*n*	%	*n*	%	*n*	%	*n*	%	*n*	%
Region																								
England	120	62	18	69	56	63	121	65	223	72	112	65	143	72	124	66	59	55	75	66	249	61	115	63
Scotland	11	6	1	4	10	11	14	8	10	3	4	2	9	4	12	6	11	10	4	4	31	8	7	4
Wales	43	22	5	19	18	20	29	16	46	15	30	17	31	16	39	21	23	22	22	19	75	19	41	22
Northern Ireland	20	10	2	8	5	6	21	11	30	10	27	16	16	8	13	7	14	13	13	11	50	12	21	11

NDNS, National Diet and Nutrition Survey Rolling Programme; PPI, proton pump inhibitor.

### General patterns of dietary vitamin B_6_ and plasma pyridoxal 5′-phosphate concentration in the UK population

**Fig. 1.**
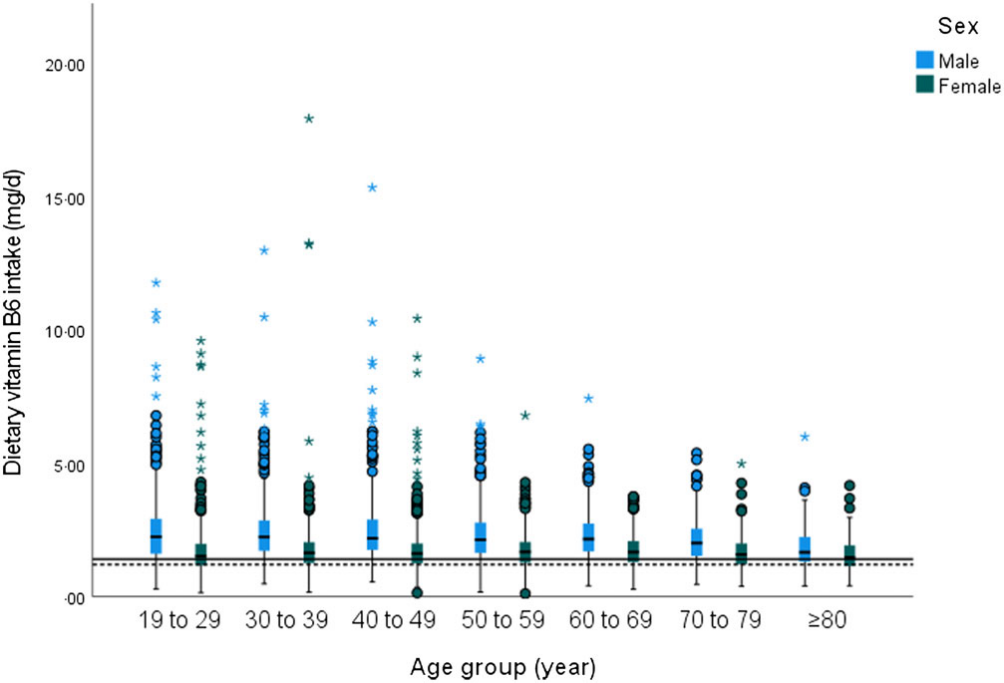
Dietary vitamin B_6_ intake among UK adults aged ≥ 19 years based on sex and age group, 2008–2017.

The median (25th–75th percentile) intakes of dietary vitamin B_6_ across the whole population (1·7 (1·4–2·3) mg/d) met the RNI for both females and males (1·2 and 1·4 mg/d respectively, Fig. 1). Moreover, males consumed more dietary vitamin B_6_ than females (2·1 (1·6–2·8) mg/d and 1·6 (1·2–2) mg/d respectively; *P* < 0·001). We found a significant association between dietary intake of vitamin B_6_, sex and age group in the total population (*P* < 0·001), using multiple linear regression analysis (online Supplementary 10), which confirmed the general trend of age-dependent reductions in dietary vitamin B_6_ in each sex (*P* < 0·001) (Table 2). Among males, significant reductions were seen in the oldest group (≥80+ group) compared with all other groups. A similar trend for females aged ≥ 80+ years was also noted with his trend becoming apparent when comparing against the 30–39 years, 50–59 years and 60–69 years groups (*P* < 0·05) (Table 2).

**Table 2.** Vitamin B_6_ intake and plasma PLP concentration among UK adults aged ≥19 years based on sex and age group, 2008–2017

Marker	Participant No.	Median	25th–75th percentiles			25th–75th percentiles	*P* value	Median (25th–75th percentiles) among age group (year)														
				Median between sex				19–29		30–39		40–49		50–59		60–69		70–79		≥ 80		*P* value
				Sex	Median			Median	25th–75th percentiles	Median	25th–75th percentiles	Median	25th–75th percentiles	Median	25th–75th percentiles	Median	25th–75th percentiles	Median	25th–75th percentiles			
Dietary vitamin B_6_ intake (mg d^–1^)	6802	1·7 *Min*: 0·1 *Max*: 18	1·4–2·3	M	2·1 *Min*:0·2 *Max*: 15·3 (*n* 2810)	1·6–2·8	<0·001	2·2 *Min*:0·3 *Max*:11·8 (*n* 363)	1·6–2·9	2·2 *Min*:0·5 *Max*:13·1 (*n* 475)	1·7–2·9	2·1 *Min*:0·6 *Max*:15·3 (*n* 566)	1·7–2·9	2·1 *Min*:0·2 *Max*:9·1 (*n* 519)	1·6–2·8	2·1 *Min*: 0·4 *Max*:7·4 (*n* 449)	1·7–2·7	2·0 *Min*:0·5 *Max*:5·4 (*n* 291)	1·5–2·5	1·6 *Min*:0·4 *Max*:6·1 (*n* 147)	1·3–2·2	<0·001
				F	1·6 *Min*: 0·1 *Max*: 17·9 (*n* 3992)	1·2–2		1·5 *Min*:0·1 *Max*:9·6 (*n* 600)	1·2–2·1	1·6 *Min*:0·2 *Max*:18·1 (*n* 721)	1·3–2	1·6 *Min*:0·1 *Max*:10·4 (*n* 793)	1·3–2·1	1·6 *Min*:0·1 *Max*:6·8 (*n* 652)	1·3–2	1·6 *Min*:0·3 *Max*:3·8 (*n* 579)	1·3–2·1	1·5 *Min*:0·4 *Max*:5·1 (*n* 423)	1·2–2·1	1·4 *Min*:0·4 *Max*:4·2 (*n* 224)	1·1–1·9	<0·001
Plasma PLP concentration (nmol l^–1^)	3281	42·8 *Min*: 6 *Max*: 602	28·6–63·9	M	47·4 *Min*: 6 *Max*: 355 (*n* 1363)	31·1–69·8	<0·001	67·3 *Min*:20 *Max*:248 (*n* 139)	46–87·4	54·3 *Min*:10 *Max*:317 (*n* 218)	39–83·4	51·5 *Min*:9 *Max*:355 (*n* 285)	35·5–71·1	48·1 *Min*:9 *Max*:241 (*n* 279)	31·6–65·4	37·5 *Min*:11 *Max*:216 (*n* 255)	27·4–60	32·1 *Min*:8 *Max*:197 (*n* 131)	20·2–53·1	28·7 *Min*:6 *Max*:104 (*n* 56)	16·8–41·2	<0·001
				F	39·9 *Min*: 7 *Max*: 602 (*n* 1918)	27·1–58·8		40·1 *Min*:7 *Max*:397 (*n* 227)	28·7–60·3	40·3 *Min*:9 *Max*:292 (*n* 312)	28–61·6	37·6 *Min*:8 *Max*:548 (*n* 434)	26·5–54·3	45·7 *Min*:8 *Max*:602 (*n* 351)	30·2–67·4	37·8 *Min*:8 *Max*:380 (*n* 319)	25·4–56·2	42·0 *Min*:7 *Max*:249 (*n* 191)	25·9–59·4	31·5 *Min*:8 *Max*:278 (*n* 84)	20·1–53·3	<0·001

PLP, pyridoxal 5' phosphate; plasma PLP concentration of < 20 nmol/l is indicative of vitamin B_6_ deficiency, while 20–<30 nmol/l is indicative of marginal deficiency. RNI for dietary vitamin B_6_ intake for adults aged 19 years and above is 1·4 mg/d for male and 1·2 mg/d for female. Mann–Whitney *U*-test was used to assess the significant differences between two categorical data, while the Kruskal–Wallis H test was used for more than two groups as described in the method. Data are expressed as median (25th–75th percentiles).

The median (25th–75th percentile) concentrations of plasma PLP across the whole population (42·8 (28·6–63·9) nmol/l) were above the cut-off of vitamin B_6_ deficiency (above the cut-off of < 20 nmol/l) (Table 2). Moreover, 90 % of total population had plasma PLP concentrations above the cut-off of < 20 nmol/l. However, 10 % showed vitamin B_6_ deficiency (plasma PLP concentration < 20 nmol/l), while 17 % of the total population had marginal vitamin B_6_ deficiency (plasma PLP concentration 20–< 30 nmol/l) (online Supplementary 1). Like dietary vitamin B_6_, females had lower median plasma PLP concentration compared with males (39·9 (27·1–58·8) nmol/l and 47·4 (31·1–69·8) nmol/l, respectively; *P* < 0·001); however, both sexes were above the cut-off of vitamin B_6_ deficiency. In fact, 12 % of female participants had vitamin B_16_ deficiency compared with 9 % of male participants (online Supplementary 1). Moreover, as shown in the regression analysis (online Supplementary 10), there was a significant association between plasma PLP concentration and age group in the total population (*P* < 0·001), but not with sex (*P* = 0·2).

**Fig. 2.**
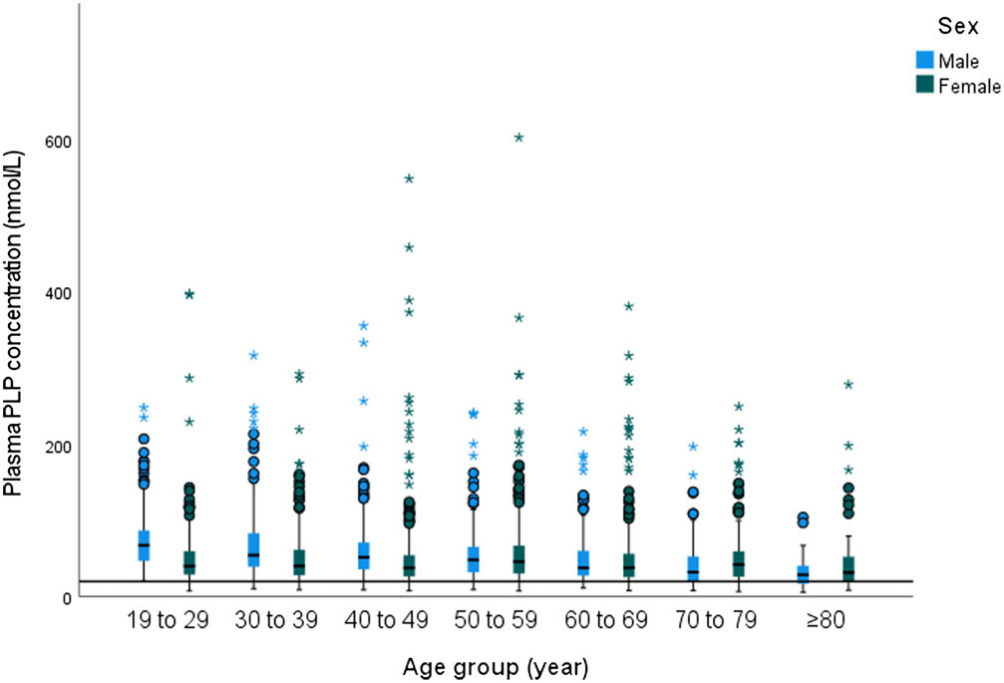
Plasma PLP concentration among UK adults aged ≥ 19 years based on sex and age group, 2008–2017. PLP, pyridoxal 5′-phosphate.

**Fig. 3.**
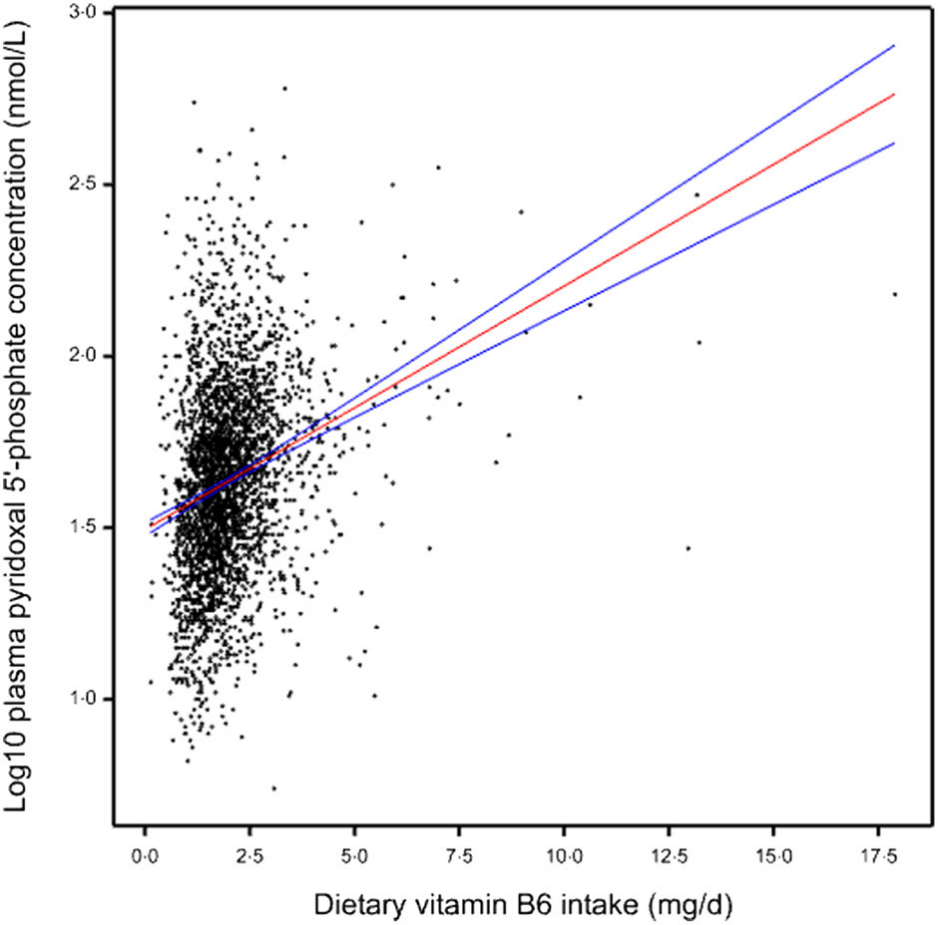
Associations between dietary vitamin B_6_ intake and plasma PLP concentration among UK adults aged ≥ 19 years based on sex and age group, 2008–2017. PLP, pyridoxal 5′-phosphate.

Also noted is a significant reduction in PLP levels with advancing age among both sexes, reaching a low of 28·7 (16·8–41·2) nmol l^–1^(*P* < 0·001) in males and 31·5 (20·1–53·3) nmol l^–1^ (*P* < 0·001) for females aged ≥ 80 years (Table 2). However, these values were still above the cut-off of deficiency (Fig. 2). Among males, a significant negative association was found between the 80+ years group and 60–69 years, 50–59 years, 40–49 years, 30–39 years, 19–29 years, *P* < 0·001. Similarly, among female aged ≥ 80+ years association was found when compared with the 50–59 years group (Table 2). A significant association between dietary vitamin B_6_ intake and log_10_ plasma PLP concentration is reported in the current analysis (R^2^ = 0·07, *P* < 0·001) (Fig. 3).

### Dietary vitamin B_6_, plasma pyridoxal 5′-phosphate level and lifestyle

#### Type of diet

Differences in plasma PLP levels had a general trend of matching specific type of diets; however, these changes were not statistically significant (Table 3). Participants who were vegetarian tended to have the lowest levels of plasma PLP concentration and dietary vitamin B_6_ intake compared with omnivore and vegan groups (36·8 (22·5–58·1) nmol l^–1^, 42·9 (28·7–64) nmol l^–1^ and 48·3 (42·8–69·3) nmol l^–1^ respectively; *P* = 0·1) and (1·4 (1·1–2) mg d^–1^, 1·7 (1·4–2·3) mg d^–1^ and 1·8 (1·4–2·2) mg d^–1^ respectively; *P* < 0·001). Of the included vegetarians, 13 % had vitamin B_6_ deficiency and 22 % had marginal deficiency compared with 10 % and 17 % of omnivores, respectively (online Supplementary 2). In the current research, no association was found after doing the regression analysis (online Supplementary 10) between diet types and dietary vitamin B intake nor plasma PLP concentrations.

**Table 3.** The impact of type of diet, smoking and alcohol consumption on vitamin B_6_ intake and plasma PLP concentration among UK adults aged ≥ 19 years

Lifestyle		Participant No.	Median of dietary vitamin B_6_ (mg d^–1^)	25th–75th percentiles	*Min*	*Max*	*P*	Participant No.	Median of plasma PLP concentration (nmol l^–1^)	25th–75th percentiles	*Min*	*Max*	*P*
Type of diet	Omnivore	6636	1·7	1·4–2·3	0·1	18	<0·001	3198	42·9	28·7–64	6	602	0·1
	Vegetarian	157	1·4	1·1–2	0·4	6·8		75	36·8	22·5–58·1	8	208	
	Vegan	9	1·8	1·4–2·2	0·6	2·9		8	48·3	42·8–69·3	31	80	
Smoking	Non-smokers	3611	1·8	1·4–2·3	0·1	17·9	<0·001	1794	46	31–67	6	548	<0·001
	1–5 cigarettes/d	249	1·7	1·3–2·2	0·1	13·2		109	41·9	23·3–71·3	9	185	
	6–9 cigarettes/d	137	1·7	1·2–2·4	0·7	6·2		52	33·1	21·6–56·1	10	230	
	≥ 10 cigarettes/d	1021	1·6	1·2–2·3	0·2	15·3		447	32·3	21·5–50	8	355	
Alcohol consumption	None-alcoholic	2714	1·6	1·2–2·2	0·1	15·3	<0·001	1211	36·8	24·9–56·9	7	397	<0·001
	≤ 4 units/d for men, ≤ 3 units/d for women	1727	1·8	1·41–2·26	0·4	18		887	44·2	29·5–44·2	6	457	
	> 4 units/d and ≤ 8 units/d for men, > 3 units/d and ≤ 6 units/d for women	1108	1·8	1·4–2·4	0·2	10·5		579	46·2	32·9–65	8	380	
	> 8 units/d for men, > 6 units/d for women	1239	2·0	1·5–2·7	0·4	13·2		600	49·7	32·9–73·1	9	602	

PLP, pyridoxal 5′ phosphate. Mann–Whitney *U*-test was used to assess the significant differences between two categorical data, while the Kruskal–Wallis H test was used for more than two groups as described in the method. Data are expressed as median (25th–75th percentiles).

#### Smoking and alcohol consumption

Results showed an association between smoking and reduction in plasma PLP concentration (*P* < 0·001) but not with dietary vitamin B_6_. Plasma PLP concentrations differed within the smoker group and were linked with the numbers of cigarettes smoked per day (Table 3). Results showed that people who smoked ≥ 10 cigarettes a day had a significantly lower plasma PLP concentration as compared with the non-smokers and those smoking fewer cigarettes per day (32·3 (21·5–50) nmol l^–1^ and 46 (31–67) nmol l^–1^ and 41·9 (23·3–71·3) nmol l^–1^ and 33·1 (21·6–56·1) nmol l^–1^, *P* < 0·001: ≥ 10 cigarettes d^–1^ and non-smoker and 1–5 cigarettes d^–1^ and 6–9 cigarettes d^–1^, respectively). We found 21 % of people who smoked ≥ 10 cigarettes d^–1^ had vitamin B_6_ deficiency compared with 6 % of non-smokers (online Supplementary 2).

A general trend showed that higher plasma PLP concentrations among people who consume higher units/d compared with non-alcoholic and lower units/d of alcohol (*P* < 0·01; Table 3). However, no association was found between alcohol and dietary vitamin B_6_ intake (online Supplementary 10).

#### Medication use

Dietary vitamin B_6_ intake and plasma PLP concentrations differed among users of medication as compared with non-users (Table 4). Among the reported medications (Table 1), dietary vitamin B_6_ levels were unaffected by most medication usage, except for those using analgesic, antidepressants, Ca blockers and diuretic and proton pump inhibitors. Users of these classes of the drug showed significant reductions in vitamin B_6_ among the users compared with the non-users (Table 4). However, regression analysis (online Supplementary 10) showed that dietary vitamin B_6_ intake was associated only with an antidepressant (*P* = 0·007).

**Table 4.** The impact of therapeutic drug use on vitamin B_6_ intake and plasma PLP concentration among UK adults aged ≥ 19 years

Lifestyle		Participant No.	Median of dietary vitamin B_6_ (mg d^–1^)	25th–75th percentiles	*Min*	*Max*	*P*	Participant No.	Median of PLP concentration (nmol l^–1^)	25th–75th percentiles	*Min*	*Max*	*P*
Medication use	Analgesic (user)	194	1·4	1·1–1·8	0·2	8·9	< 0·001	112	31·5	20·2–48·4	8	175	< 0·001
	Analgesic (non-user)	1260	1·6	1·2–2·1	0·4	11·8		847	41·8	28·5–62·1	8	388	
	Antibacterial (user)	26	1·7	1·3–2·2	1	6·8	0·1	16	32	21·9–43·4	10	147	0·1
	Antibacterial (non-user)	1428	1·5	1·2–2·1	0·2	11·8		943	41·3	27·4–59·5	8	388	
	Antidiabetic (user)	89	1·5	1·1–1·9	0·6	3·4	0·07	54	26·2	19·1–42	9	286	< 0·001
	Anti-diabetic (non-user)	1365	1·5	1·2–2·1	0·2	11·8		905	41·7	28·4–61	8	388	
	Anti-depressant (user)	185	1·4	1–1·9	0·2	9	< 0·001	114	33·9	22·1–57	8	257	0·008
	Anti-depressant (non-user)	1269	1·6	1·2–2·1	0·4	11·8		845	41·4	28·6–59·7	8	388	
	ACE inhibitor (user)	309	1·6	1·3–2	0·4	6·4	0·3	195	38·2	23·3–57·1	7	257	0·1
	ACE inhibitor (non-user)	2149	1·6	1·3–2·2	0·1	18		1460	41·4	28–60·8	8	388	
	Beta-blocker (user)	173	1·5	1·6–2·1	0·4	3·9	0·1	88	32·8	22·5–53	8	240	0·013
	Beta-blocker (non-user)	2285	1·6	1·3–2·2	0·1	18		1567	41·4	28·1–60·5	7	388	
	Ca blocker (user)	199	1·5	1·2–2	0·6	18	0·021	124	32·5	21·3–50·3	9	240	< 0·001
	Ca blocker (non-user)	2259	1·6	1·3–2·2	0·1	13·2		1531	41·5	28·4–61·6	7	388	
	Diuretic (user)	188	1·5	1·2–1·9	0·4	4·1	0·008	115	32·2	20·4–55·2	7	198	< 0·001
	Diuretic (non-user)	2270	1·6	1·3–2·2	0·1	18		1540	41·5	28·4–60·8	8	388	
	Antiplatelet (user)	107	1·6	1·2–2	0·4	3·1	0·3	64	28·9	19·6–54·8	8	240	0·004
	Anti-platelet (non-user)	1347	1·5	1·2–2·1	0·2	11·8		895	41·5	28·4–59·8	8	388	
	Asthma prescribed (user)	114	1·5	1·2–2	0·4	9·1	0·5	69	31·3	20·1–45·6	8	261	0·001
	Asthma prescribed (non-user)	1340	1·5	1·2–2·1	0·2	11·8		890	41·4	28·1–60·8	8	388	
Medication use	Lipid lowering (user)	405	1·5	1·3–2·1	0·4	6·4	0·4	245	36·6	22·1–52·5	7	257	< 0·001
	Lipid lowering (non-user)	2053	1·6	1·3–2·1	0·1	18		1410	41·6	28·7–62·1	8	388	
	Proton pump inhibitor (user)	184	1·5	1·1–1·9	0·4	6·8	0·023	107	32·8	23–57	8	240	0·018
	Proton pump inhibitor (non-user)	1270	1·6	1·2–2·1	0·2	11·8		852	41·5	28·2–9·7	8	388	

PLP, pyridoxal 5' phosphate. Mann–Whitney *U*-test was used to assess the significant differences between two categorical data, while the Kruskal–Wallis H test was used for more than two groups as described in the method. Data are expressed as median (25th–75th percentiles).

Moreover, almost all the medication users, except antibacterial and ACE inhibitor groups, showed significant reductions in plasma PLP levels compared with the non-users (*P* ≤ 0·01: Table 4). Among the reported twelve medications, we found that seven drugs were associated with reduced plasma PLP concentrations which are analgesics, antibacterial, antidiabetics, antidepressant, Ca blockers, prescribed asthma and lipid-lowering drugs (*P* < 0·001, *P* = 0·04, *P* = 0·002, *P* = 0·03, *P* = 0·03, *P* = 0·003 and *P* = 0·01, respectively; online Supplementary 10), which confirmed the general trend of plasma PLP reduction among the medication users. Importantly, the percentage of participants who had vitamin B_6_ deficiency was higher in all the medication users compared with non-users (online Supplementary 3).

## Discussion

Evidence from epidemiological and animal studies reports that diminished vitamin B_6_ intake and plasma PLP concentrations are associated with negative health outcomes in humans^(38–43)^. The current study sought to utilise the available NDNS datasets to determine dietary vitamin B_6_ intake and plasma PLP concentration in free-living adults, representative of the general UK population (≥ 19 years). In general, most participants met the RNI for vitamin B_6_ intake and were above the cut-off of vitamin B_6_ deficiency (plasma PLP concentrations were above 20 nmol/l). However, 10 % of the total population had vitamin B_6_ deficiency, while 17 % had marginal vitamin B_6_ deficiency. A noted tendency for both dietary vitamin B_6_ intake and plasma PLP concentration was that these tended to decrease with age and also with lifestyle factors such as smoking and medication usage.

When the data were stratified for age, a significant difference in vitamin B_6_ intake and plasma PLP concentrations in each sex was seen (Table 2). The age-related declines in both dietary vitamin B_6_ intake and plasma PLP concentration (Table 2) previous parallel observations in the elderly^(44,45)^ and changes documented in the current research indicate reductions in both vitamin B_6_ intake and plasma PLP concentration across age groups (*P* < 0·001) (Table 2). Moreover, we reported significant associations between plasma PLP concentration, dietary vitamin B_6_ and age group (*P* < 0·001).

The significance of these findings is highlighted by the knowledge that low dietary vitamin B_6_ intakes in the elderly are associated with frailty^(46)^, impaired mobility risk reduction (OR = 0·66;^(47)^), physical performance^(48)^, increased serum homocysteine levels^(49)^, cognitive impairment^(50)^ and immune function impairment^(51)^. Moreover, a recent meta-analysis revealed an inverse correlation between dietary vitamin B_6_ and CVD (*P* < 0·05;^(52)^). Therefore, long-term, suboptimal levels of vitamin B_6_ intake could predispose individuals to various health risks. Similarly, low plasma PLP levels predispose to a higher risk of venous thrombosis^(53)^, myocardial infarction^(54)^, diabetes mellitus^(55)^, COVID-19 infection^(56)^, oxidative stress^(38)^, increasing risk of cancer in diabetic patients^(57,58)^, immune system disruption and CRP level elevations^(39)^ that suggests increased risk of inflammation conditions^(58)^. These associations indicate the importance of both dietary vitamin B_6_ intake and plasma PLP concentration in age-related health conditions (reviewed in Parra and colleagues^(24)^). While it is acknowledged that the participants in the current research are free living UK population, the observed declines in dietary vitamin B_6_ intake and plasma PLP concentration maybe more significant in those members of the general population suffering from chronic ill health, reduced food intakes or those residing in care homes^(59)^. Clearly, given the important roles of these molecules in ageing, stress response, drug metabolism and the immune system^(60)^, further research is warranted. To date, a small number of human intervention studies have assessed vitamin B_6_ supplementation on health, and these show that increased vitamin B_6_ intakes is beneficial to health^(4,61,62)^.

Few human clinical trials have been reported that assess vitamin B_6_ or associated metabolites ( https://clinicaltrials.gov/ct2/results?term=Pyridoxal+5%27Phosphate&Search=Search), of these, most are combinational treatments of PLP alongside various therapeutics making interpretation difficult. Few studies have assessed PLP supplementation alone in the elderly to manage age-related health conditions. While all participants in the current work still meet their RNI for vitamin B_6_ intake and are above the cut-off of < 20 nmol/l of plasma PLP concentration, levels are diminished with age and this could pose potential health risks, and further studies are needed.

Lifestyle factors such as smoking, alcohol consumption and therapeutic drug usage were common among participants in the current work. We found an association between smoking and reduction in plasma PLP concentrations (*P* < 0·001) but not with dietary vitamin B_6_. In the smoking groups, levels of plasma PLP decreased with increased cigarette usage (10+ per day), with 21 % of people who smoke ≥ 10 cigarettes d^–1^ having plasma PLP levels below the cut-off of vitamin B_6_ deficiency. A similar result to that reported by Jain and Lim^(63)^. While data are sparse, the possible reasons for this are proposed increases in the activity of PLP-dependent enzymes such as alanine phosphatase and reduction in the levels of serum albumin, both common in smokers^(64)^.

Furthermore, positive association between plasma PLP concentration and alcohol is reported. We found the non-alcohol-consuming participants had the lowest dietary vitamin B_6_ compared with people who drink alcohol, although no association was found between alcohol and dietary vitamin B_6_ intake in this study. Van Der Gaag and colleagues^(65)^ found serum PLP levels were reportedly increased by 17 %, 15 % and 30 % following the consumption of three servings of wine, spirits and beer over 3 weeks alcohol consumption period. This could be due to the fact that beer contains vitamin B_6_^(65)^. In our study, beer drinkers had higher dietary vitamin B_6_ intake and plasma PLP concentration than non-drinkers (online Supplementary 5 showed vitamin B_6_ intake and plasma PLP concentration among different types of alcoholic beverages in the NDNS dataset).

Moreover, in people following different types of diets, like vegetarians and vegans, no significant differences in plasma PLP levels were noted in these groups. This may be reflective of diet diversity or supplementation^(66)^ (online Supplementary 4).

The most significant observation made in the current research was the impact of medication on vitamin B_6_ intakes and plasma PLP levels. Interestingly, it is noteworthy that the number of prescription drugs taken in this population increased with advancing age (online Supplementary 6). This finding correlates with previous studies showing on average that therapeutic drug use is stratified as follows 50–60 years (2 drugs)^(32)^, 60–70 years (3–4 drugs)^(67)^ and 70+ years (4–10 drugs)^(68)^. Twelve drug groups were identified based on therapeutic drug use, ranging from analgesics to proton pump inhibitor (as shown in Table 1). It was noteworthy that there were associations between reductions in plasma PLP concentrations and usage of analgesics, antibacterial, antidiabetics, antidepressant, calcium blockers, asthma prescriptions and lipid-lowering drugs. Low dietary vitamin B_6_ intake was only associated with an antidepressant. Other medications have previously been reported to diminish vitamin B_6_ status, such as antiepileptic^(5)^ and oral contraceptives^(69)^. While reduced levels of plasma PLP concentrations among the medication users are above the threshold of vitamin B_6_ deficiency (above 20 nmol/l of plasma PLP concentration) (Table 4), these drug groups could be impacting on susceptible individuals such as the elderly. Indeed, long-term use of medications is common among aged populations to manage long-term conditions and one could speculate that polypharmacy in these groups could be driving nutrient deficiencies and in turn health complications^(33)^. Among the elderly, aged 65–79 years, vitamin B_6_ intakes are positively associated with handgrip strength^(48)^ and physical performance^(70)^. In fact, Kjeldby and colleagues^(44)^ found that PLP levels were normal among all elderly people (mean age of 85·3 years) who used vitamin B_6_ supplements as compared with non-users having a deficiency. Among the elderly, vitamin B_6_ supplements were seen to improve immune responses post 2 months of a 50 mg/d of vitamin B_6_ supplementation^(71)^. Similarly, vitamin B_6_ supplementation (20 mg/d for three months) was seen to improve memory health in the elderly^(72)^.

Collectively, the current work highlights the provision of the NDNS dataset for the assessment of health and nutrient-related interactions. The current research found important links between lifestyle factors and vitamin B_6_ intake and plasma PLP levels, which are important for health. Mitigation of vitamin B_6_ deficiency and targeting people at risk of nutritional deficiency could help in reducing health-related comorbidities. UK adults (≥ 19 years) met RNI of vitamin B_6_ intake and were above the cut-off of vitamin B_6_ deficiency; however, a common pattern that emerged from this study was the tendency for both vitamin B_6_ intake and PLP concentrations to decrease with age and lifestyle factors such as smoking and medication usage. Importantly, medication usage frequently showed a negative impact on plasma PLP levels in humans. This information could be an important consideration in the nutritional assessment of the elderly, particularly those that smoke or individuals using polypharmacy. However, it must be acknowledged that the limitations of the current findings are the lack of information relating to dosage and duration of drug use in the participants and the timing between food diaries and blood samples in the NDNS. Another limitation of the current work is that the available data on vitamin B status does not take into account the metabolic turnover of vitamin B_6_ in the respective population. Past research indicates that plasma PLP concentration measures are robust markers of vitamin B_6_ status; however, they may not reflect the total subcellular compartmentalisation of vitamin B_6_. Clearly, further work is needed in this field of research.

## Supplementary material

For supplementary material accompanying this paper visit https://doi.org/10.1017/S0007114523000417. click here to view supplementary material
