# Supplementary material for: Impact of lifestyle factors on dietary vitamin B6 intake and plasma pyridoxal 5′-phosphate level in UK adults: National Diet and Nutrition Survey Rolling Programme (NDNS) (2008–2017)
Source: Br J Nutr. 2023 Feb 15;130(8):1403–15. doi: 10.1017/S0007114523000417 (PMC10511679; doi:10.1017/S0007114523000417)
Supplement: Supplementary file 1 [file S0007114523000417sup001.docx]

**Supplementary 1**. Number and percentage of participants depending on the cut-off value of plasma PLP concentration

among UK adults aged ≥19 years based on sex and age group, 2008-2017.

| Plasma PLP concentration  (nmol L^-1^) | All participants | Between sex | | Between age in each sex group | | | | | | |
| --- | --- | --- | --- | --- | --- | --- | --- | --- | --- | --- |
|  |  |  |  | 19-29 | 30-39 | 40-49 | 50-59 | 60-69 | 70-79 | ≥80 |
| Above the cut-off of vitamin B6 deficiency | 2937 (90%) | **M** | 1247 (91%) | 138 (11%) | 211 (17%) | 274 (22%) | 257 (20%) | 234 (19%) | 100 (8%) | 33 (3%) |
|  |  | **F** | 1690 (88%) | 209 (12%) | 277 (16%) | 381 (23%) | 318 (19%) | 274 (16%) | 168 (10%) | 63 (4%) |
| Marginal vitamin B6 deficiency * | 558 (17%) | **M** | 196 (14%) | 10 (5%) | 21 (11%) | 36 (18%) | 34 (17%) | 60 (31%) | 29 (15%) | 6 (3%) |
|  |  | **F** | 362 (19%) | 45 (12%) | 55 (15%) | 86 (24%) | 51 (14%) | 72 (20%) | 33 (9%) | 20 (6%) |
| Vitamin B6 deficiency** | 344 (10%) | **M** | 116 (9%) | 1 (1%) | 7 (6%) | 11 (9%) | 22 (19%) | 21 (18%) | 31 (27%) | 23 (20%) |
|  |  | **F** | 228 (12%) | 18 (8%) | 35 (15%) | 53 (23%) | 33 (15%) | 45 (20%) | 23 (10%) | 21 (9%) |

**Abbreviations**: (*): plasma PLP concentration is 20-<30 nmol L^-1^: indicative for marginal vitamin B6 deficiency, (**): plasma PLP concentration is <20 nmol L^-1^:

indicative of vitamin B6 deficiency. Percentages represent the total population, however, when stratified by age, the percentages are sup-group percentages.

**Supplementary 2**. Number and percentage of participants depending on the cut-off value of plasma PLP concentration

among UK adults aged ≥19 years based on diet type, smoking and alcohol consumption, 2008-2017.

| Lifestyle | | Plasma PLP concentration (nmol L^-1^) | | |
| --- | --- | --- | --- | --- |
|  |  | Above the cut-off of vitamin B6 deficiency | Marginal vitamin B6 deficiency * | Vitamin B6 deficiency** |
| Diet type | Omnivore | 2864 (90%) | 542(17%) | 334 (10%) |
|  | Vegetarian | 65 (87%) | 16 (22%) | 10 (13%) |
|  | Vegan | 8 (100%) | 0 | 0 |
| Smoking | None-smokers | 1684 (94%) | 259 (14%) | 110 (6%) |
|  | 1-5 cigarettes/d | 91 (83%) | 19 (17%) | 18(17%) |
|  | 6-9 cigarettes/d | 43 (83%) | 13 (25%) | 9(17%) |
|  | ≥10 cigarettes/d | 354 (79%) | 106 (24%) | 93(21%) |
| Alcohol consumption | None-alcoholic | 1025 (85%) | 249 (21%) | 186 (15%) |
|  | ≤4 units/d for men, ≤3 units/d for women | 798 (90%) | 140 (16%) | 89 (10%) |
|  | >4 units/d and ≤8 units/d for men, >3 units/d and ≤6 units/d for women | 541 (93%) | 76 (13%) | 38(7%) |
|  | > 8 units/d for men, > 6 units/d for women | 569 (95%) | 92 (15%) | 31(5%) |

Abbreviations: (*): plasma PLP concentration is 20-<30 nmol L^-1^: indicative for marginal

vitamin B6 deficiency, (**): plasma PLP concentration is <20 nmol L^-1^: indicative of

vitamin B6 deficiency.

**Supplementary 3.** Number and percentage of participants depending on the cut-off value of plasma PLP concentration

among UK adults aged ≥19 years based on therapeutic drug, 2008-2017.

| Lifestyle | | Plasma PLP concentration (nmol L^-1^) | | |
| --- | --- | --- | --- | --- |
|  |  | Above the cut-off of vitamin B6 deficiency | Marginal vitamin B6 deficiency * | Vitamin B6 deficiency** |
| Medication use | Analgesic  (user) | 85  (76%) | 26  (23%) | 27  (24%) |
|  | Analgesic  (non-user) | 751  (89%) | 139  (17%) | 96  (11%) |
|  | Antibacterial (user) | 13  (81%) | 4  (25%) | 3  (19%) |
|  | Antibacterial (non-user) | 823  (87%) | 161  (17%) | 120  (13%) |
|  | Antidiabetic (user) | 39  (72%) | 17  (31%) | 15  (28%) |
|  | Antidiabetic (non-user) | 797  (88%) | 148  (16%) | 108  (12%) |
|  | Antidepressant (user) | 88  (77%) | 26  (23%) | 26  (23%) |
|  | Antidepressant (non-user) | 748  (88%) | 139  (16%) | 97  (12%) |

**Supplementary 3**. Number and percentage of participants depending on the cut-off value of plasma PLP concentration

among UK adults aged ≥19 years based on therapeutic drug, 2008-2017 (continue).

| Lifestyle | | PLP (nmol L^-1^) | | |
| --- | --- | --- | --- | --- |
|  |  | Above the cut-off of vitamin B6 deficiency | Marginal vitamin B6 deficiency * | Vitamin B6 deficiency** |
| Medication use | ACE inhibitor (user) | 162  (83%) | 31  (16%) | 33  (17%) |
|  | ACE inhibitor (non-user) | 1280  (88%) | 243  (17%) | 180  (12%) |
|  | Beta blocker (user) | 70  (79%) | 23  (26%) | 18  (21%) |
|  | Beta blocker (non-user) | 1372  (88%) | 251  (16%) | 195  (12%) |
|  | Ca blocker (user) | 97  (78%) | 31  (25%) | 27  (22%) |
|  | Ca blocker (non-user) | 1345  (88%) | 243  (16%) | 186  (12%) |
|  | Diuretic  (user) | 88  (77%) | 26  (23%) | 27  (23%) |
|  | Diuretic  (non-user) | 1354  (88%) | 248  (16%) | 186  (12%) |
|  | Antiplatelet (user) | 45  (70%) | 14  (22%) | 19  (30%) |
|  | Antiplatelet (non-user) | 791  (88%) | 151  (17%) | 104  (12%) |

**Supplementary 3**. Number and percentage of participants depending on the cut-off value of plasma PLP concentration

among UK adults aged ≥19 years based on therapeutic drug, 2008-2017 (continued).

| Lifestyle | | PLP (nmol L^-1^) | | |
| --- | --- | --- | --- | --- |
|  |  | Above the cut-off of vitamin B6 deficiency | Marginal vitamin B6 deficiency * | Vitamin B6 deficiency** |
|  | Asthma prescribed (user) | 52  (75%) | 16  (23%) | 17  (25%) |
|  | Asthma prescribed  (non-user) | 784  (88%) | 149  (17%) | 106  (12%) |
|  | Lipid lowering (user) | 192  (78%) | 47  (19%) | 53  (22%) |
|  | Lipid lowering (non-user) | 1250  (89%) | 227  (16%) | 160  (11%) |
|  | Proton pump inhibitor  (user) | 89  (83%) | 27  (25%) | 18  (17%) |
|  | Proton pump inhibitor  (non-user) | 747  (88%) | 138  (16%) | 105  (12%) |

Abbreviations: (*): plasma PLP concentration is 20-<30 nmol L^-1^: indicative for marginal

vitamin B6 deficiency, (**): plasma PLP concentration is <20 nmol L^-1^: indicative of

vitamin B6 deficiency.

**Supplementary 4**. Proportion of people who took multivitamin supplements based on diet type: (n=582).

| Diet type | Number of participants (%) |
| --- | --- |
| Omnivore | 553 (95%) |
| Vegetarian | 26 (4%) |
| Vegan | 3 (1%) |

**Supplementary 5**. Alcoholic beverages participants consumed in the NDNS data set among UK adults aged ≥19 years.

| Alcohol drink | Participant No. | Median (^25^th – ^75^th percentile) of Dietary vitamin B6 (mg day^-1^) | *Min* | *Max* | *P*  value | Participant No. | Median (^25^th – ^75^th percentile) of Plasma PLP (nmol L^-1^) | *Min* | *Max* | *P* value |
| --- | --- | --- | --- | --- | --- | --- | --- | --- | --- | --- |
| Strong beer | Drinker:  234 | 2.1 (1.5-2.8) | 0.5 | 8.6 | <0.001 | 113 | 47.4 (33.9-67.9) | 6 | 200 | 0.4 |
|  | Non-drinker: 3847 | 1.8 (1.4-2.4) | 0.1 | 17.7 |  | 1954 | 46.2 (31.1-66.8) | 8 | 602 |  |
| Normal beer | Drinker: 1451 | 2.2 (1.6-2.9) | 0.3 | 12.9 | <0.001 | 738 | 47.6 (32.8-72.1) | 8 | 355 | 0.04 |
|  | Non-drinker: 2630 | 1.8 (1.4-2.2) | 0.2 | 17.9 |  | 1329 | 45.5 (30.6-65) | 6 | 602 |  |
| Wine  (drinker) | 1942 | 1.7 (1.4-2.2) | 0.3 | 17.9 | <0.001 | 1016 | 47.2 (32.3-67.2) | 8 | 602 | 0.09 |
| Wine  (non-drinker) | 2139 | 1.9 (1.4-2.6) | 0.1 | 13.2 |  | 1051 | 45.1 (30.5-66.7) | 6 | 355 |  |
| Spirits (drinker) | 991 | 1.8 (1.3-2.3) | 0.1 | 13.2 | <0.001 | 484 | 44.9 (29.1-64.7) | 6 | 602 | 0.1 |
| Spirits  (non-drinker) | 3090 | 1.9 (1.4-2.4) | 0.3 | 17.9 |  | 1583 | 46.8 (32.1-67.5) | 8 | 457 |  |

**Supplementary 6**. Number of prescribed medications in the NDNS data set among UK adults aged ≥19 years based on age groups.

| Medication; n (%) | Age (years) | | | | | | |
| --- | --- | --- | --- | --- | --- | --- | --- |
|  | 19-29 | 30-39 | 40-49 | 50-59 | 60-69 | 70-79 | ≥80 |
| Analgesic (n=194 out of 1454) | 5 (3%) | 16 (8%) | 23 (12%) | 42 (22%) | 47 (24%) | 39 (20%) | 22 (11%) |
| Antibacterial (n=26 out of 1454) | 2 (8%) | 2 (8%) | 3 (12%) | 6 (22%) | 5 (19%) | 5 (19%) | 3 (12%) |
| Antidiabetic (n=89 out of 1454) | 5 (6%) | 3 (3%) | 15 (17%) | 14 (16%) | 23 (26%) | 17 (19%) | 12 (13%) |
| Antidepressant (n=185 out of 1454) | 16 (9%) | 15 (8%) | 42 (23%) | 38 (20%) | 42 (23%) | 24 (13%) | 8 (4%) |
| ACE inhibitor (n=309 out of 2458) | 1 (0.3%) | 7 (2%) | 19 (6%) | 67 (21.7%) | 92 (30%) | 82 (27%) | 41 (13%) |
| Beta blocker (n=173 out of 2458) | 3 (2%) | 2 (1%) | 10 (6%) | 29 (16%) | 49 (28%) | 46 (27%) | 34 (20%) |
| Ca blocker (n=199 out of 2458) | 0 | 3 (1%) | 16 (8%) | 32 (16%) | 63 (32%) | 53 (27%) | 32 (16%) |
| Diuretic  (n=188 out of 2458) | 0 | 1 (1%) | 4 (2%) | 27 (14%) | 48 (26%) | 64 (34%) | 44 (23%) |
| Antiplatelet (n=107 out of 1454) | 0 | 0 | 5 (5%) | 9 (8%) | 33 (31%) | 34 (32%) | 26 (24%) |
| Asthma prescribed (n=114 out of 1454) | 9 (8%) | 8 (7%) | 18 (16%) | 25 (22%) | 29 (25%) | 14 (12%) | 11 (10%) |
| Lipid lowering (n=405 out of 2458) | 0 | 3 (1%) | 19 (4%) | 73 (18%) | 144 (36%) | 113 (28%) | 53 (13%) |
| Proton pump inhibitor (n=184 out of 1454) | 2 (1%) | 9 (5%) | 17 (9%) | 38 (21%) | 37 (20%) | 48 (26%) | 33 (18%) |

**Supplementary 7.** Plasma pyridoxic acid (PA) concentration among UK adults aged ≥19 years based on sex and age group, 2008-2017.

| Marker | Participant No. | Median  (25^th^ – 75^th^ percentile) | Median (25^th^ – 75^th^ centile) between sex | | *P* value | Median (25^th^ – 75^th^ percentile) among age group (year) | | | | | | | |
| --- | --- | --- | --- | --- | --- | --- | --- | --- | --- | --- | --- | --- | --- |
|  |  |  |  |  |  | 19-29 | 30-39 | 40-49 | 50-59 | 60-69 | 70-79 | ≥80 | *P* value |
| Plasma PA (nmol L^-1^) | 3281 | 23.2 (16.8-32.4)  *Min*: 5.1  *Max*: 730.7 | M | 24.7 (18.4-33.7)  *Min*: 5.1  *Max*:730.7  (**n**=1363) | <0.001 | 23.7 (16.2-32.7)  *Min*: 8.3  *Max*:101.1  (**n**=139) | 24.2 (17.6-31.3)  *Min*: 5.5  *Max*:207.3  (**n**=218) | 22.5 (17.4-30.4)  *Min*: 8.7  *Max*:125.4  (**n**=285) | 24.5 (19.2-31.6)  *Min*: 5.1  *Max*:100.4  (**n**=279) | 25.6 (18.5-36)  *Min*: 7.5  *Max*:205.7  (**n**=255) | 30.4 (21.9-41.8)  *Min*: 6.2  *Max*:115.4  (**n**=131) | 29.1 (21.8-42.3)  *Min*: 9.3  *Max*:730.7  (**n**=56) | <0.001 |
|  |  |  | F | 21.8 (15.7-31.2)  *Min*: 5.7  *Max*:536.2  (**n**=1918) |  | 18.8 (13.9-24.9)  *Min*: 6.1  *Max*:282.6  (**n**=227) | 18.9 (14.1-26.3)  *Min*: 7.1  *Max*:355  (**n**=312) | 19.6 (14.3-27.1)  *Min*: 5.7  *Max*:234  (**n**=434) | 23.1 (17.4-32.1)  *Min*: 8.1  *Max*:536.2  (**n**=351) | 24.0 (17.3-3)  *Min*: 6.7  *Max*:446.5  (**n**=319) | 29.7 (21-40.9)  *Min*: 9  *Max*:406.4  (**n**=191) | 30.7 (20.7-47.9)  *Min*: 9.4  *Max*:266.1  (**n**=84) | <0.001 |

**PA**, plasma pyridoxic acid. Mann–Whitney *U*‐test was used to assess the significant differences between two categorical data, while the Kruskal–Wallis H test used for more than two groups as descried in the method: Data are expressed as median (25^th^ – 75^th^ percentile ).

**Supplementary 8.** The impact of type of diet, smoking and alcohol consumption on plasma PA concentrations

among UK adults aged ≥19 years.

| Lifestyle | | Participant No. | Median (25^th^ – 75^th^ percentile) of PA concentration (nmol L^-1^) | *Min* | *Max* | *P* |
| --- | --- | --- | --- | --- | --- | --- |
| Type of diet | Omnivore | 3198 | 23.2 (16.8-32.4) | 5.1 | 730.7 | 0.001 |
|  | Vegetarian | 75 | 18.7 (14.4-27.5) | 6.7 | 84.8 |  |
|  | Vegan | 8 | 33.6 (30.6-41.3) | 21.8 | 43.6 |  |
| Smoking | 1-5 cigarettes/d | 109 | 17.9 (14-28.3) | 5.7 | 355 | 0.7 |
|  | 6-9 cigarettes/d | 52 | 17.2 (13.4-25) | 8 | 70.2 |  |
|  | ≥10 cigarettes/d | 447 | 18.4 (13.8-25.9) | 5.1 | 406.4 |  |
| Alcohol consumption | ≤4 units/d for men, ≤3 units/d for women | 887 | 24.2 (17.9-34.7) | 6.9 | 730.7 | 0.022 |
|  | >4 units/d and ≤8 units/d for men, >3 units/d and ≤6 units/d for women | 579 | 23.1 (17.2-31) | 6.4 | 245.9 |  |
|  | > 8 units/d for men, > 6 units/d for women | 600 | 23.6 (17.5-31.1) | 5.1 | 446.5 |  |

**PA**, plasma pyridoxic acid. Mann–Whitney *U*‐test was used to assess the significant differences between two categorical

data, while the Kruskal–Wallis H test used for more than two groups as descried in the method: Data are expressed

as median (25^th^ – 75^th^ percentile).

**Supplementary 9.** The impact of therapeutic drug use on plasma PA concentrations among UK adults aged ≥19 years.

| Lifestyle | | Participant No. | Median (25^th^ – 75^th^ percentile) of PA concentration (nmol L^-1^) | *Min* | *Max* | *P* |
| --- | --- | --- | --- | --- | --- | --- |
| Medication use | Analgesic  (user) | 112 | 21.9  (15.2-29.8) | 6.7 | 355 | 0.5 |
|  | Analgesic  (non-user) | 847 | 22.1  (16.2-31.6) | 6 | 446.5 |  |
|  | Antibacterial (user) | 16 | 19.1  (15.2-29.7) | 11.2 | 85 | 0.4 |
|  | Antibacterial (non-user) | 943 | 22.1  (16.1-31.5) | 6 | 446.5 |  |
|  | Antidiabetic (user) | 54 | 24.6  (16.3-35.9) | 9.6 | 195.9 | 0.2 |
|  | Antidiabetic (non-user) | 905 | 22.0  (16.1-31.1) | 6 | 446.5 |  |
|  | Antidepressant (user) | 114 | 19.3  (14.2-31.9) | 6.7 | 355 | 0.08 |
|  | Antidepressant (non-user) | 845 | 22.4  (16.3-31.4) | 6 | 446.5 |  |
|  | ACE inhibitor (user) | 195 | 26.2  (19.60-37.10) | 7.3 | 406.4 | <0.001 |
|  | ACE inhibitor (non-user) | 1460 | 21.6  (15.7-30.3) | 5.1 | 730.7 |  |
|  | Beta blocker (user) | 88 | 28.7  (18.4-41.2) | 5.1 | 266.1 | <0.001 |
|  | Beta blocker (non-user) | 1567 | 21.9  (16-30.7) | 5.7 | 730.7 |  |

**Supplementary 9.** The impact of therapeutic drug use on plasma PA concentrations among UK adults aged ≥19 years (continued).

| Lifestyle | | Participant No. | Median (25^th^ – 75^th^ percentile) of PA concentration (nmol L^-1^) | *Min* | *Max* | *P* |
| --- | --- | --- | --- | --- | --- | --- |
| Medication use | Ca blocker (user) | 124 | 24.0  (19.1-34.4) | 5.1 | 266.1 | 0.007 |
|  | Ca blocker (non-user) | 1531 | 21.9  (15.9-31.1) | 5.7 | 730.7 |  |
|  | Diuretic  (user) | 115 | 27.2  (19.6-38.2) | 5.1 | 406.4 | <0.001 |
|  | Diuretic  (non-user) | 1540 | 21.8  (15.8-30.6) | 5.7 | 730.7 |  |
|  | Antiplatelet (user) | 64 | 29.0  (21.3-41.6) | 10.4 | 406.4 | <0.001 |
|  | Antiplatelet (non-user) | 895 | 21.8  (15.8-30.5) | 6 | 446.5 |  |
|  | Asthma prescribed (user) | 69 | 21.8  (14.9-30.1) | 8.7 | 406.4 | 0.5 |
|  | Asthma prescribed  (non-user) | 890 | 22.1  (16.1-31.5) | 6 | 446.5 |  |
|  | Lipid lowering (user) | 245 | 25.3  (18.2-36.1) | 7.3 | 406.4 | <0.001 |
|  | Lipid lowering (non-user) | 1410 | 21.6  (15.6-30.3) | 5.1 | 730.7 |  |
|  | Proton pump inhibitor  (user) | 107 | 24.2  (17.9-39) | 6.4 | 406.4 | 0.014 |
|  | Proton pump inhibitor  (non-user) | 852 | 21.8  (15.9-30.6) | 6 | 446.5 |  |

**Supplementary 10.** Associations of plasma PLP concentration and dietary vitamin B6 with lifestyle factors using linear regression analysis

| Variable | Plasma PLP concentration | | Dietary vitamin B6 intake | |
| --- | --- | --- | --- | --- |
|  | ***P* value** | **R^2^** | ***P* value** | **R^2^** |
| Age | <0.01 | 0.12 | <0.01 | 0.12 |
| Sex | 0.2 | 0.12 | <0.01 | 0.12 |
| Male: +age | <0.01 | 0.12 | <0.01 | 0.02 |
| Female: +age | <0.01 | 0.01 | <0.01 | 0.003 |
| Type of diet | 0.3 | 0.06 | 0.2 | 0.12 |
| Smoking | <0.01 | 0.11 | 0.3 | 0.13 |
| Alcohol | <0.01 | 0.08 | 0.9 | 0.14 |
| Analgesics | <0.01 | 0.18 | 0.9 | 0.16 |
| Antibacterial | 0.05 | 0.08 | 0.6 | 0.15 |
| Antidiabetic | <0.01 | 0.08 | 0.7 | 0.15 |
| Antidepressant | 0.03 | 0.08 | <0.01 | 0.15 |
| ACE inhibitor | 0.7 | 0.06 | 0.3 | 0.13 |
| Beta blocker | 0.3 | 0.07 | 0.1 | 0.14 |
| Ca blocker | 0.03 | 0.07 | 0.3 | 0.15 |
| Diuretic | 0.09 | 0.07 | 0.1 | 0.13 |
| Antiplatelet | 0.2 | 0.07 | 0.3 | 0.15 |
| Asthma prescribed | <0.01 | 0.07 | 0.3 | 0.14 |
| Lipid lowering | 0.01 | 0.06 | 0.4 | 0.14 |
| Proton pump inhibitor | 0.4 | 0.07 | 0.9 | 0.15 |
